# Supplementary material for: ConNIS and labeling instability: New statistical methods for improving the detection of essential genes in TraDIS libraries
Source: PLoS Comput Biol. 2026 Mar 6;22(3):e1013428. doi: 10.1371/journal.pcbi.1013428 (PMC12991369; doi:10.1371/journal.pcbi.1013428)
Supplement: Attachment — Submitted filename: Response to Reviewers.pdf [file pcbi.1013428.s008.pdf]

Rebuttal letter for “ConNIS and labeling instability: new statistical methods for improving the detection of essential genes in TraDIS libraries”

We sincerely thank the reviewers for their thoughtful, constructive, and encouraging evaluation of our work. We appreciate the suggestions regarding potential extensions of our analysis and improvements to the manuscript’s readability.

In the revised manuscript, all additions are highlighted in blue and all deletions are crossed out and highlighted in red. We have added four supplementary files, including (i) a gene-wise classification comparison for each real-world dataset and (ii) a proof-of-concept analysis illustrating a classification beyond the binary “essential vs. non-essential” distinction. In addition, we updated Theorem 2 and its proof in Supplementary File 1 by a more stringent formulation.

a. **Replies to Reviewer #1**

Reviewer #1:

“1. The demonstrated performance of your method is highly compelling. Given its advantages, the research community would benefit from the systematic re-analysis of existing public TraDIS data. I strongly recommend that you apply your pipeline to all relevant datasets in the DEG database. Generating a unified, high-confidence set of essential gene predictions across multiple organisms and studies using your superior method would be an invaluable resource. This would allow for more accurate comparative genomics and hypothesis generation. I encourage you to make this comprehensive prediction set easily accessible, ideally through a dedicated web portal or a repository, alongside the publication of this manuscript.”

Authors’ response:

We thank the reviewer for this insightful suggestion and for highlighting the potential impact of our method. We fully agree that a systematic re-analysis of relevant TraDIS datasets curated in the Database of Essential Genes (DEG) to generate cross-organism predictions of potentially essential genes would be highly valuable for the community. Such an effort could substantially strengthen the statistical support for essentiality annotations and foster comparative genomics and hypothesis generation.

However, we believe that this task warrants a substantial research project of its own and goes beyond the scope and objectives of the present methodological work. In particular, a comprehensive re-analysis is currently constrained by significant data availability and standardization issues. The DEG database curates study-level results (gene lists) but does not generally host the raw sequencing data or insertion-site maps. For many curated studies, raw reads are not deposited in public repositories

or are no longer accessible. Moreover, heterogeneity in experimental protocols, sequencing depth, insertion density, and often sparse metadata for older studies would require extensive, manual, case-by-case curation to ensure valid inputs for a unified re-analysis.

We therefore regard the reviewer's suggestion as an important avenue for future work, ideally as a community effort that builds on our software. In the revised manuscript, we now explicitly mention this perspective in the Discussion (last paragraph).

Reviewer #1:

"*2. To further strengthen the manuscript and highlight the biological relevance of your methodological improvements, a more detailed comparative analysis is needed. Specifically, I request a focused section that identifies discrepancies. Please provides a list or table of specific genes for the organisms analyzed where your new predictions of essentiality conflict with the predictions from the original studies. For each major discrepancy, offers a plausible and detailed explanation in biological context. Please explain the gene's function and why the new prediction makes biological sense. This analysis will move beyond simply stating that your method is "better" and will provide concrete, biologically-grounded examples of its value."*

Authors' response:

We agree with the reviewer that a closer inspection of gene-wise discrepancies between methods will improve the comparison and better illustrate the practical impact of ConNIS. In response, we have taken the following steps:

- **Comprehensive discrepancy lists:** For all three real-data examples, we now provide the complete list of genes for which at least one of the methods classifies a gene as "essential" as supplementary files (Supplementary Files 4, 5 and 6). These tables allow readers to examine in detail where ConNIS and competing methods agree or disagree.
- **New section "Biological relevance":** We have added a dedicated section entitled "Biological relevance" to the Results, in which we focus on examples of "major discrepancies", defined as genes for which the ConNIS classification differs from that of at least four of the five competitor methods. For these genes, we describe the gene function (as far as annotated), summarize relevant literature where available, and explain why the classification provided by ConNIS is biologically plausible.
- **Short genes and classification power:** In this section, we also emphasize that ConNIS retains classification power for shorter genes which are often excluded or down-weighted in other approaches. We believe these in-depth examples strengthen our claim that ConNIS, together with the instability criterion, constitutes a competitive and robust methodology for essentiality analysis.

We hope that these additions address the reviewer's request and make the advantages of ConNIS more tangible from a biological perspective.

Reviewer #1:

"*3. The binary classification of genes into "essential" and "non-essential" is a known oversimplification. As famously demonstrated by the JCVI-syn3.0 minimal genome project, a third category—quasi-essential genes—is critical for robust growth and fitness. I suggest evaluating the performance of statistical methods on the quasi-essential gene set, and incorporating relevant discussions."*

Authors' response:

We fully agree that gene essentiality is not strictly binary and that quasi-essential genes play an important role in robust growth and fitness, particularly when time and environmental conditions are considered. In our view, the "basic" or "initial" essentiality analyzed in this work corresponds to the minimal set of genes required for survival under a given condition at a fixed time point. Our main contribution is to provide a statistically rigorous method for these binary calls, in close analogy to the state-of-the-art methods used as benchmarks in this work. We do not claim that this fully captures the spectrum of gene functions or fitness effects.

At the same time, we share the reviewer's view that extending our method toward the classification of quasi-essential and fitness-related genes is highly desirable. Doing this in a statistically rigorous way would require an explicit stochastic model describing how insertion sites and read counts evolve over time and across conditions, including selection, genetic drift, and bottlenecks. For example, Mahmutovic et al. (2020, https://doi.org/10.1016/j.csbj.2020.03.021) show that drift and realistic bottlenecks in Tn-Seq experiments can cause random extinction of viable mutants, which may lead to misclassification if analyses rely solely on simple fold-change criteria.

In the landmark JCVI-syn3.0 minimal genome project mentioned by the reviewer, "essential", "non-essential", and "quasi-essential" genes are defined using explicit but heuristic rules for insertion patterns at two passages. In brief, genes without insertions, or with only sparse insertions near the gene ends, are classified as "essentia"l; genes with many insertions at both the initial passage (P0) and after several passages (P4) are classified as "non-essential"; and genes that are frequently hit at P0 but not at P4 are classified as "quasi-essential", with further subdivision based on the severity of the growth defect. While the rule that genes "not hit at all, or sparsely hit in the terminal 20% of the 3′ end" are classified as "essential" is intuitive, the approach relies on fixed thresholds for insertion-free regions. Changing these thresholds (e.g. 15% or 10%, at the beginning and/or end of the gene) would alter which genes are labeled "essential" and, consequently, which genes are labeled "quasi-essential". Critically, no stochastic model is provided to justify the specific choice of these thresholds, and the approach does not explicitly model stochastic processes such as selection, drift, and bottlenecks across time.

In response to the reviewer's suggestion, we have implemented the following:

- **Discussion of graded essentiality:** We now explicitly discuss graded "essentiality", "quasi-essential" genes, and fitness-like effects in the Discussion section, clarifying how our current framework fits into this broader picture.
- **Proof-of-concept analysis (new supplement):** We have added a proof-of-concept analysis as Supplementary File 7. In this analysis, we apply ConNIS to a relatively low-density transposon library of *Ruegeria pomeroyi* and show how ConNIS, combined with the instability approach, yields a **continuous gene-wise essentiality evidence score** based on Shannon information. We use this score to classify "quasi-essential" genes as those that are "non-essential" labeled in the initial library but classified "essential" after growth over time (see pages 4–5 of the supplement for results and visualizations). We further compare these ConNIS-based measures to derived fitness scores and relate them to previously reported fitness estimates for the same system.

This proof-of-concept analysis demonstrates that ConNIS is compatible with a three-class view of essentiality (non-essential, quasi-essential, essential) and with continuous fitness effects. We view this as a starting point for future work that combines our analytic solution for insertion-free gaps with a fully specified stochastic model for the time-dependent distribution of insertion sites.

b. Replies to Reviewer #2

We thank Reviewer #2 for the careful reading and the positive overall assessment of our methodological work.

Reviewer #2:

"However, although the mathematical formulation is rigorous, the article may prove challenging to follow for scientists without an advanced statistical background. In addition, some sections are overly technical, which may hinder comprehension for a broader biological audience."

Authors' response:

Thank you for this important remark. We appreciate your recognition of the rigor of our mathematical formulation, and we also share your concern regarding accessibility for readers with diverse backgrounds.

In response, we have:

- Revised the **Methods** and **Results** sections to include additional interpretive sentences alongside the main formulas, explicitly describing their purpose and intuition.
- Rephrased several technical passages to improve readability and to make the logic of each methodological step clearer.
- Focused more on the major results by removing some plots (see also Reviewer #3 comments).
- Updated **Figure 2** (the schematic explanation of the instability criterion) by inserting h^{sub} to be consistent with the text.

Our aim is to maintain the level of rigor required for computational scientists while making the manuscript more approachable and informative for biologists, who are also a key audience of this journal. We believe these revisions significantly improve readability, and we hope the manuscript is now more accessible to a broader readership.

Reviewer #2:

As a future recommendation; acknowledging that it may fall beyond the scope of the present publication—although results are compared with E. coli and Salmonella libraries, the study focuses almost exclusively on statistical performance metrics (MCC, PRC). It would be desirable to include experimental validation, for instance, verification of predicted essential genes through knockout experiments or phenotypic analyses.
Overall, the work is methodologically robust and experimentally convincing, combining an innovative statistical framework with extensive validation. It represents a significant contribution to TraDIS data analysis, particularly under experimental conditions characterised by sparse or uneven insertion densities. Nevertheless, its impact could be further enhanced by including direct experimental validation and by making the presentation more accessible to researchers without a quantitative background.

Authors' response:

We thank the reviewer for this thoughtful forward-looking suggestion and for the very positive overall assessment of our work.

We fully agree that experimental validation of genes labeled "essential" (for instance via targeted knockouts or phenotypic analyses) would provide an additional and biologically compelling assessment of our method's performance. At the same time, we concur with the reviewer that such experiments would go beyond the scope of this methodological paper, and would require substantial additional experimental work and resources.

In line with Reviewer #2's recommendation and with Reviewer #1's request, we have taken steps to connect our statistical results more explicitly to biological interpretation:

- We have added the new **"Biological relevance"** section (see response to Reviewer #1, Comment 2), in which we illustrate and interpret major discrepancies between ConNIS and competitor methods for multiple example genes, discussing available functional annotations and literature where possible.
- By focusing on such examples, we provide biologically grounded illustrations of the statement that ConNIS differs from existing methods, which we hope is especially helpful for non-statistical readers.

Together with the revisions aimed at improving accessibility (see Comment 1 above), we believe these changes move the manuscript toward the direction suggested by the reviewer, while keeping the scope consistent with a methods-focused paper.

c. Replies to Reviewer #3

We thank Reviewer #3 for the careful reading of the manuscript and for pointing out several minor issues.

Reviewer #3:

"*Figure 3,4,5: There are a lot of similar plots. Could you keep the smallest and largest number of IS/samples , or the most interesting ones, and put the rest in supplemental data maybe ?*

Authors' response:

We agree that focusing on the most informative plots improves the readability of the manuscript. In the revised version, we have streamlined the figures as follows:

- For the **synthetic data**, we now show only the most relevant settings in the main figures: a medium-to-high density setting, a low-density setting, and a cold-spot setting.
- For the **semi-synthetic data**, we show the low- and high-density settings in the main text and have moved additional scenarios to the supplementary material.

We believe this restructuring helps readers appreciate the main performance differences between methods without being overwhelmed by similar-looking plots, while still providing full details in the supplement for interested readers.

Reviewer #3:

Table 1: Do you have a way to compare the MCC you obtained with tuning with those obtained with the previous methods, such as the ones used in the papers cited in the paragraph from line 54 to 69?"

We agree that it is important to relate the MCC values achieved with our instability-based tuning approach to those obtained using parameter settings that are commonly used in previous TraDIS studies.

In the revised manuscript, we therefore added an explicit comparison between:

1. **"Literature-oriented" parameter choices**, i.e. values that are representative of those used in prior publications;
2. The **instability-tuned** parameter values used in our benchmarks; and
3. The **optimal MCC** found over a grid of parameter values for each method.

We updated Table 1 accordingly and expanded the corresponding text in the Results section to describe these comparisons.

More specifically:

- For the **Exp. vs. Gamma** approach, we now report results for $\log_2(4)$ and $\log_2(12)$ thresholds, which have been used previously for relatively low- or high-density, respectively, libraries and in comparative studies.
- For **InsDens**, we used the parameter combinations employed in the benchmarks of the original publication.
- For the **Binomial** and **Tn5Gaps** models, we considered their original untuned form (i.e. without our weighting factor *w*) alongside the instability-tuned versions.

We believe that these additional comparisons strengthen our argument for using the instability criterion to select threshold/parameter values a priori and to improve transparency and comparability across studies.
