# Supplementary material for: ConNIS and labeling instability: New statistical methods for improving the detection of essential genes in TraDIS libraries
Source: PLoS Comput Biol. 2026 Mar 6;22(3):e1013428. doi: 10.1371/journal.pcbi.1013428 (PMC12991369; doi:10.1371/journal.pcbi.1013428)
Supplement: Attachment — Submitted filename: Response_to_Reviewers_auresp_2.pdf [file pcbi.1013428.s009.pdf]

# Second rebuttal letter for “ConNIS and labeling instability: new statistical methods for improving the detection of essential genes in TraDIS libraries”

We sincerely thank the reviewer for the second review. In response to the suggestion, we performed additional analyses beyond the real-world examples included in the manuscript. We provide the resulting resource in a citable Zenodo repository and refer to this new analysis in the revised manuscript (highlighted in green). In addition, we also corrected a typo in Formula (1) where the rounding symbol for the weighted expected number of insertions was missing and explained the idea of using the “Vandermonde identity” in Theorem 3 in Supplement 1.

a. **Replies to Reviewer #1**

   *Reviewer #1:*

   *“I am pleased with the authors' substantial revisions, especially the new biological analysis section. However, regarding my first major suggestion, I find the decision to defer all systematic re-analysis to future work to be a missed opportunity that diminishes the potential impact of this otherwise excellent method.*

   *While I appreciate the authors' concerns about data heterogeneity, their argument about data inaccessibility is somewhat overstated. Many high-quality Tn5 TraDIS studies have deposited their raw reads in public repositories. A more impactful and feasible approach would be to include a focused demonstrative analysis. Specifically, I strongly recommend that the authors select a curated set (e.g., 5-10) of publicly available Tn5 TraDIS datasets from the SRA and apply their ConNIS pipeline to generate a unified prediction set.*

   *Providing this curated resource – either as a supplementary table or through a citable data repository – would transform this manuscript from a methods description into a valuable community resource. Given that the authors have already developed a complete, publicly available analysis pipeline, this task should be quite feasible. I believe this addition would significantly enhance the manuscript's utility and should be incorporated prior to publication.”*

   Authors’ response:

   We thank the reviewer for the constructive suggestion to increase the manuscript’s utility as a community resource. In line with this recommendation, we performed an additional demonstrative re-analysis of publicly available Tn5-based TraDIS/Tn-Seq datasets and compiled a harmonized output resource.

Specifically, we curated and analyzed eight additional independent published datasets (six bacterial and two archaeal organisms) that were publicly accessible in different forms, ranging from raw FASTQ reads to pre-computed insertion-site tables. For each study, we derived insertion-site positions (or used author-provided insertion-site lists), applied ConNIS using the same general decision framework, and reported the resulting putative essential-gene lists. These additional results have been made publicly available via a Zenodo repository (https://doi.org/10.5281/zenodo.18538449), which is referenced in the revised manuscript at the end of the Discussion. We hope this curated set addresses the reviewer's request for a focused and practically useful demonstration across multiple studies.

We also wish to clarify the scope of ConNIS with respect to "end-to-end" processing. ConNIS is provided as a ready-to-use R package that takes as input (i) insertion-site positions and (ii) a gene annotation table (gene name/locus tag, start, stop). As it is common for essentiality-calling methods, ConNIS is designed to be agnostic to upstream processing from raw sequencing reads, because accurate insertion-site mapping typically requires multiple study-dependent bioinformatic steps (e.g., read filtering, trimming, mapping, and quality control) and depends on study-specific transposon design and library construction. In practice, these steps are commonly performed with established toolchains such as TRANSIT or Bio-TraDIS, frequently in combination with study-specific parameter choices and additional scripts.

By providing the curated multi-study results on Zenodo and documenting the per-study processing in the accompanying materials, we aim to (i) give readers a concrete, reproducible demonstration on public data, (ii) provide a set of putative essential genes across multiple organisms and (iii) make it straightforward for others to apply ConNIS to additional datasets using their preferred upstream mapping pipeline.

*Reviewer #1:*

*Additionally, I note that the reference list contains formatting problems. Several entries (including refs 4, 8, 9, 10, 11, 12, and 15) are missing the required article numbers. The entire reference list should be carefully reviewed and corrected to meet journal formatting standards.*

Authors' response:

We thank the reviewer for the careful inspection of the reference list. We have systematically reviewed and corrected the entire reference list, including the entries highlighted by the reviewer, and added missing article numbers and other metadata as required by the journal's formatting guidelines.
